# Novel Molecular Type of *Clostridium difficile* in Neonatal Pigs, Western Australia

**DOI:** 10.3201/eid1905.121062

**Published:** 2013-05

**Authors:** Michele M. Squire, Glen P. Carter, Kate E. Mackin, Anjana Chakravorty, Torbjörn Norén, Briony Elliott, Dena Lyras, Thomas V. Riley

**Affiliations:** The University of Western Australia, Nedlands, Western Australia, Australia (M.M. Squire, B. Elliott, T.V. Riley);; Monash University, Clayton, Victoria, Australia (G.P. Carter, K.E. Mackin, A. Chakravorty D. Lyras);; Örebro University Hospital, Örebro, Sweden (T. Norén);; PathWest Laboratory Medicine, Nedlands (T.V. Riley)

**Keywords:** Clostridium difficile, C. difficile, Cdiff, pigs, piglets, domestic, zoonoses, animals, veterinary, enteritis, diarrhea, ribotyping, PCR, prevalence, sequence analysis, DNA, Australia, bacteria

## Abstract

*Clostridium difficile* causes neonatal enteritis in piglets; strains of PCR ribotype 078 are most commonly identified. We investigated *C. difficile* prevalence in piglets in Australia and isolated a novel strain with a unique pathogenicity locus. In a mouse infection model, this strain produced more weight loss than did a ribotype 078 strain.

*Clostridium difficile* is the causative agent of severe enteritis (“scouring”) in neonatal piglets 1–7 days of age throughout Canada, the United States, and Europe (*1*). Although deaths attributable to *C. difficile* infection (CDI) generally are low because of good stockmanship, piglets that survive CDI remain 10%–15% underweight on average and take additional time to wean (*2*).

Colonization frequency of *C. difficile* in scouring piglets is as high as 52%; this rate declines to 4% by 2 months of age (*3*). *C. difficile* is also commonly found in feces from apparently healthy piglets, which contributes to environmental contamination. Widespread air and surface contamination of the piggery environment with *C. difficile*, presumably in the form of long-surviving spores, may play a role in the epidemiology of CDI in pigs and subsequent community-acquired infection in humans (*4*). 

In Europe and the United States, the genotypes of *C. difficile* isolates that cause disease in humans and production animals overlap, particularly PCR ribotype 078, which predominates in pigs worldwide. This ribotype is increasing in prevalence and associated with severe community-acquired CDI in humans geographically located near pig farms (*5*). *C. difficile* has also been found in retail food, including meat products, seafood, and vegetables (*6*). 

*C. difficile* in piglets in Australia has not been systematically investigated, despite reports of idiopathic enteritis nationwide. It is likely that that *C. difficile* strains in piglets in Australia are different from those found in the rest of the world because of Australia’s geographic isolation, strict quarantine laws regarding importation of livestock, and low human population and pig density. We studied *C. difficile* prevalence in scouring neonatal piglets and evaluated a novel strain of *C. difficile* isolated from these piglets by using multiple identification methods.

## The Study

Rectal swab specimens were collected during July–November 2009 from 185 neonatal piglets on 3 farms that were experiencing scouring problems. The farms were located at 2 geographic locations in Western Australia (20 km apart) and were owned by a commercial farrow-to-finish operation. At the time of the study, 50%–80% of litters were scouring, with death rates of 11%–14%. The sick piglets had early-onset, nonhemorrhagic, yellow, pasty-to-watery diarrhea; disease course without treatment was ill-thrift, anorexia, dehydration, and death. Healthy piglets were treated prophylactically at 1–3 days of age with amoxicillin or penicillin. 

Of the 185 piglets, 131 were on 2 farms at the same geographic location that had the most severe scouring problems. The remaining 54 piglets were on a high biosecurity farm at a separate location with a variable scouring problem; 11 of these animals were asymptomatic. Test results for *Escherichia coli*, rotavirus, and *C. perfringens* were negative for all animals; we did not test for porcine reproductive and respiratory syndrome virus or transmissible gastroenteritis coronavirus because they are considered exotic (i.e., no reported outbreaks) in Australia.

Samples were cultured directly onto cycloserine cefoxitin fructose agar and incubated anaerobically at 37°C for 48 h. The swabs were then inoculated into a Robertson’s cooked-meat selective enrichment broth and incubated anaerobically at 37°C for 7 days; spores were then selected by alcohol shock (1:1 with anhydrous ethanol). The spores were then cultured onto cycloserine cefoxitin fructose agar with 0.1% taurocholic acid added. Putative *C. difficile* colonies were subcultured onto prereduced blood agar and identified by Gram stain, characteristic colony morphology, and smell.

Toxin profiling was by PCR detection of the toxin A (*tcdA*), toxin B (*tcdB*), and binary toxin (*cdt*) genes (*7*,*8*). Isolates underwent PCR ribotyping and were compared with human reference *C. difficile* strains from Australia and international ribotypes from the Anaerobe Reference Laboratory (Cardiff, Wales, UK) (*9*). Genome shotgun sequencing of a representative isolate (designated strain AI35) was performed by using the Illumina HiSeq2000 platform (Australian Genome Research Facility, The Walter and Eliza Hall Institute of Medical Research, Parkville, Victoria, Australia). Paired-end reads of 31,085,914 bp were assembled into 117 contigs by using the Velvet software suite (*10*).

Toxin B quantitation was performed by using a Vero cell cytotoxicity assay (*11*). Vero cells were exposed to serial 2-fold dilutions of *C. difficile* cell-free culture supernatants from a ribotype 027 human strain (M7404), a ribotype 078 animal strain (JGS6133), and strain AI35. *C. difficile* strain 630, a recognized low-toxin producer, was included as a control. After the cultures were incubated overnight at 37°C in 5% CO_2_, morphologic changes were examined by microscopy. The endpoint was scored as the last dilution at which 100% cytopathic effect was observed. Assays were performed in triplicate on independent culture supernatants.

In vivo virulence was assessed by using a mouse model of CDI (*12*). Specifically, 6–8-week-old male C57/B6 mice (5 mice per strain) were force-fed 1 × 10^7^ spores of the same *C. difficile* strains used in the Vero cell assays. Mice were housed in separate cages to avoid cross-contamination and monitored daily for weight loss and signs of disease.

Of the 185 piglets tested, *C. difficile* was isolated from 114 (62%): 70 (53%) of 131 piglets from the herds with severe scouring, 33 (77%) of 43 piglets from the herd with variable scouring, and 11 (100%) of 11 asymptomatic piglets. Isolates were clonal; all were novel PCR ribotype 237 and had a toxin profile of *tcd*A^–^*tcd*B^+^*cdt*A^+^*cdt*B^+^. 

**Figure 1:**
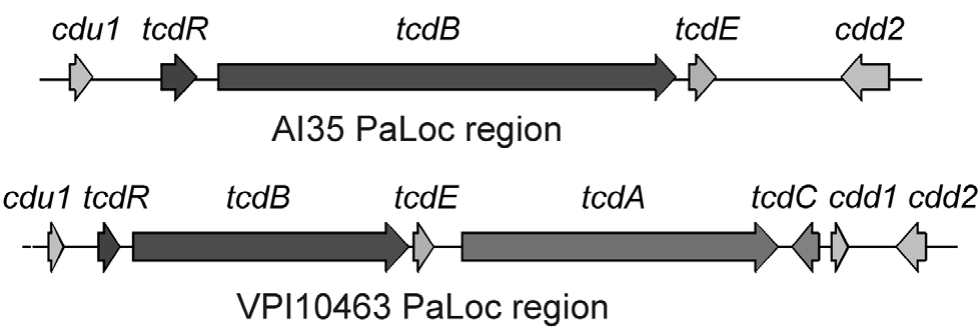
Structure of pathogenicity locus (PaLoc) and flanking regions in *Clostridium difficile* strains AI35 and VPI10463. Boxes indicate open reading frames; arrows indicate direction of transcription. Encoded genes are indicated above the arrows. Figure not drawn to scale.

Genome sequencing of strain AI35 showed a novel pathogenicity locus (PaLoc) structure (Figure 1). A large deletion had removed the *tcdA* and *tcdC* genes and a portion of the adjacent *cdd1* gene located outside the PaLoc. Strain AI35 also encoded a variant TcdE. The AI35 binary toxin locus was complete and contained an intact copy of *cdtR*, unlike ribotype 078 isolates, which encode a *cdtR* with a premature stop codon. Strain AI35 may therefore be a more proficient binary toxin producer than ribotype 078 strains. Despite these variations, multilocus sequence typing showed that strain AI35 belongs to the same clade (clade 5) and sequence type (11) as ribotype 078 strains [strain AI15 in the report by Stabler et al. (*13*) is the same ribotype as AI35].

**Figure 2:**
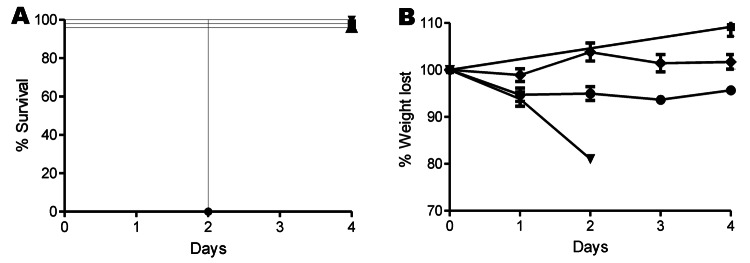
A) Survival and B) percentage of weight lost in mice over 4 days after infection with *Clostridium difficile*. Male C57/B6 mice were infected with *C. difficile* spores for strains M7404 (triangles), JGS6133 (078) (diamonds), or A135 (circles); phosphate-buffered saline (squares) was used as control. Error bars in panel B indicate SEM.

In vitro testing showed AI35 produced ≈25-fold less toxin B than did the ribotype 027 (p = 0.0354 by *t* test) and ribotype 078 (p = 0.0074 by *t* test) isolates, but AI35 showed similar toxin levels to the low toxin producing strain 630. AI35-mediated cytopathic effect on Vero cells was similar to that reported for the lethal toxin of *C. sordellii* and *C. difficile* strain 8864, a toxin A^–^B^+^ human strain with mutations affecting its glucosylation substrate specificity (*14*). Strain M7404 (ribotype 027) was significantly more virulent than strain AI35 (p = 0.0001 by log-rank [Mantel Cox] test) and strain JGS6133 (p = 0.0002 by log-rank [Mantel Cox] test). All mice infected with strain M7404 died (Figure 2, panel A), but mice infected with strains AI35 and JGS6133 survived (Figure 2, panel A). Still, despite low toxin production, AI35 caused significantly greater weight loss in mice than did the ribotype 078 strain JGS6133 (p = 0.0011 by analysis of variance) (Figure 2, panel B). This difference may be the result of production of a variant toxin; similar toxins were 8-fold more toxic to mice than was toxin B produced by strain VPI10463 (*15*).

## Conclusions

Our results show that a toxigenic *C. difficile* strain circulating in piglets in Australia is of a different ribotype, 237, than that commonly found in other parts of the world. The strain we found contained a unique PaLoc and produced more weight loss in mice than did the more common ribotype 078 animal strain. Identifying this strain is the first step in detecting and responding to this emerging disease in piglets in Australia. Future studies in swine will focus on nationwide prevalence, laboratory detection, and epidemiologic investigation to understand the transmission cycle in pigs and any relationship between animal and human disease.
